# 2808. Evaluation of a Weighted-Incidence Syndromic Combination Antibiogram (WISCA) to guide empiric therapy in pneumonia cases

**DOI:** 10.1093/ofid/ofad500.2419

**Published:** 2023-11-27

**Authors:** Erik Skoglund, Leslie Banuelos, Destiny Farihi

**Affiliations:** Western University of Health Sciences, College of Pharmacy, Los Angeles, California; Western University College of Pharmacy, Los Angeles, California; Western University College of Pharmacy, Los Angeles, California

## Abstract

**Background:**

Empiric antibiotic selection must balance the need for prompt active coverage against the risk of antibiotic resistance pressure imposed by using broad agents. The guidance available from conventional antibiograms is limited in this setting as they provide no information about the relative frequency of pathogens for a given infectious syndrome. Weighted-Incidence Syndromic Combination Antibiograms (WISCAs) overcome this limitation. To better promote appropriate empiric antibiotic use at our institution, we sought to develop a WISCA depicting empiric activity of various regimens against previous cases of bacterial pneumonia.

**Methods:**

Patients admitted between 2018-2022 were screened for respiratory cultures with positive bacterial growth and a discharge diagnosis code consistent with bacterial pneumonia. Patients were excluded if they lacked radiologic evidence or clinical symptoms. The first episode of pneumonia per admission was considered. Interpretations were determined using CLSI breakpoints, and assumptions were used to extrapolate the activity of agents for which no breakpoints or MICs were available. A case was considered susceptible to a given regimen if all cultures in that case were covered by at least one agent in the regimen. The expected performance of WISCA-directed empiric therapy was compared to the activity of actual empiric therapy received by a retrospective cohort of pneumonia patients.

**Results:**

The WISCA was built using 674 respiratory cultures from 441 unique patient cases. The most common empiric agents received in the retrospective cohort were vancomycin (158, 77%), piperacillin-tazobactam (133, 65%), ceftriaxone (77, 38%), and meropenem (57, 28%). Among this cohort, 126 (61%) of patients received active empiric coverage against all case pathogens. According to the WISCA, the combination of piperacillin-tazobactam + levofloxacin provides active empiric coverage for 63% of cases, which is not significantly different than that achieved in the retrospective cohort while avoiding use of carbapenems or vancomycin.

**Conclusion:**

Our findings suggest that WISCAs provide additional guidance for empiric antibiotic prescribing compared to conventional antibiograms alone.

**Disclosures:**

**All Authors**: No reported disclosures

